# TIAM2S Mediates Serotonin Homeostasis and Provokes a Pro-Inflammatory Immune Microenvironment Permissive for Colorectal Tumorigenesis

**DOI:** 10.3390/cancers12071844

**Published:** 2020-07-08

**Authors:** Ya-Ling Chan, Wei-Chung Lai, Jia-Shing Chen, Joseph Ta-Chien Tseng, Pei-Chin Chuang, Jonathan Jou, Chung-Ta Lee, H. Sunny Sun

**Affiliations:** 1 Department of Biotechnology and Bioindustry Sciences, National Cheng Kung University, Tainan 701, Taiwan; yealingjan@yahoo.com.tw (Y.-L.C.); tctseng@mail.ncku.edu.tw (J.T.-C.T.); 2 Institute of Molecular Medicine, College of Medicine, National Cheng Kung University, Tainan 701, Taiwan; workman_pum250@hotmail.com; 3 School of Medicine for International Students, I-Shou University, Kaohsiung 840, Taiwan; cjshing77@gmail.com; 4 Department of Medical Research, Kaohsiung Chang Gung Memorial Hospital, Kaohsiung 833, Taiwan; pcjchuang@gmail.com; 5 Department of Surgery, University of Illinois, College of Medicine at Peoria, Peoria, IL 61605, USA; jjou3@uic.edu; 6 Department of Pathology, College of Medicine, National Cheng Kung University, Tainan 701, Taiwan; lcta@mail.ncku.edu.tw

**Keywords:** T-cell lymphoma invasion and metastasis 2, chronic inflammation, serotonin, T lymphocyte, inflammatory bowel disease

## Abstract

The short isoform of human TIAM2 has been shown to promote proliferation and invasion in various cancer cells. However, the roles of TIAM2S in immune cells in relation to tumor development have not been investigated. To characterize the effects of TIAM2S, we generated TIAM2S-overexpressing mouse lines and found that aged TIAM2S-transgenic (TIAM2S-TG) developed significantly higher occurrence of lymphocytic infiltration and tumorigenesis in various organs, including colon. In addition, TIAM2S-TG is more sensitized to AOM-induced colon tumor development, suggesting a priming effect toward tumorigenesis. In the light of our recent findings that TIAM2S functions as a novel regulator of cellular serotonin level, we found that serotonin, in addition to Cox2, is a unique inflammation marker presented in the colonic lesion sites in the aged TG animals. Furthermore, our results demonstrated that ectopic TIAM2S altered immunity via the expansion of T lymphocytes; this was especially pronounced in CD8^+^ T cells in combination with CXCL13/BCA-1 pro-inflammatory chemokine in the serum of TIAM2S-TG mice. Consequently, T lymphocytes and B cells were recruited to the lesion sites and stimulated IL-23/IL17A expression to form the tertiary lymphoid organs. Collectively, our research suggests that TIAM2S provokes a pro-inflammatory immune microenvironment permissive to colorectal tumorigenesis through the serotonin-induced immunomodulatory effects.

## 1. Introduction

Human T-cell lymphoma invasion and metastasis 2 (TIAM2), a homolog of TIAM1, is located on chromosome 6q25.2 and encodes two transcripts, the long form, TIAM2 (TIAM2L) (NM_012454.3, also known as TIAM2 variant 1), and the short form, TIAM2S (NM_001010927.2, also known as TIAM2 variant 2). Human TIAM1 is known to be involved in Ras or Wnt-induced tumor growth [1,2] and is associated with cancer malignancy and poorer prognosis in hepatocellular carcinoma, nasal pharyngeal carcinoma, and lymphocytic leukemia [3,4,5]. We previously demonstrated that TIAM2S was overexpressed in a majority (86%) of hepatocellular carcinoma (HCC) samples, and overexpression of TIAM2S promotes epithelial-to-mesenchymal transition (EMT), resulting in the proliferation and invasion of liver cancer cells [6]. Furthermore, TIAM2 has been shown to promote cell invasion and motility in non-small-cell lung cancer [7] and regulate cell growth of adult T-cell leukemia/lymphoma (ATL) [8]. These data indicate that aberrant expression of TIAM2S likely contributes to human malignancies.

Recently, we found that TIAM2S is normally produced in the neurons of restricted brain regions and uncovered its non-oncogenic functions as a novel regulator of serotonin level, brain neuroplasticity, and locomotion behavior [9]. Furthermore, we identified that SP1 binds directly to the GC box located in the TIAM2S core promoter and controls TIAM2S ectopic expression in liver cancer cells [10]. Interestingly, mouse homolog of human TIAM2 has been shown to have various isoforms, and the expression of Tiam2s was suggested to be controlled by its own promoter as well [11]. Unlike TIAM2L, human TIAM2S protein constitutes a Dbl homology (DH) domain and a pleckstrin homology (PH) domain and does not contain N-terminal PH-CC-EX, RBD, and PDZ domains or myristoylation sites, suggesting a lack of protein–protein interactions and membrane translocation [6]. As TIAM2S may be unable to play a role like classical GEF proteins, it is unclear how TIAM2S acts and cooperates to alter cellular dynamics and trigger tumorigenesis.

The immune system is a key determinant of the tumor microenvironment and plays critical roles in cancer development and progression [12]. Furthermore, inflammatory responses play pivotal roles at various stages of tumor development, starting from initiation through tumor promotion, all the way to metastatic progression. In fact, it is known that chronic inflammation increases cancer risk, and many types of immune and inflammatory cells are frequently present within tumors [13]. The productions of factors including cytokines and chemokines from the immune cells act in an autocrine and paracrine manner to affect tumor microenvironment and promote tumor growth. The relationship between inflammation and tumorigenesis is well-documented by cases of colon cancer in the setting of inflammatory bowel disease (IBD) and is considered a risk factor involved in sporadic and heritable colon cancer [14].

It is important to reveal the fundamental mechanism of tumorigenesis under an immune-competent microenvironment to understand how cancer cells escape immune surveillance to develop strategies for immunotherapy [15]. The roles of serotonin in modulating peripheral immunity [16,17] and cancers progression have gradually emerged [18]; thus, serotonin is considered as a key to control the host immune microenvironment. In light of our recent findings that TIAM2S functions as a novel regulator of cellular serotonin level, it is of interest to elucidate the interaction between TIAM2S and serotonin and to study the impact of this interaction on tumorigenesis. In the current study, we established transgenic mice overexpressing human TIAM2S to serve as an animal model for investigating the mechanism of TIAM2S-mediated tumorigenesis in great detail. The pathological assessment indicated that overexpression of TIAM2S in mice results in increases of spontaneous tumor incidence and tissue inflammation frequency. Furthermore, TIAM2S overexpression sensitized animals to grow carcinogen-induced tumors upon exposure. We found TIAM2S expression mediates the expansion of CD8+ T lymphocytes and colonic inflammation through the interleukin (IL)-23/IL-17 axis. In addition, we identified that serotonin-facilitated chronic inflammation is the mechanism behind observed TIAM2S-mediated tumorigenesis. Collectively, our study demonstrated that TIAM2S modulates serotonin homeostasis to cause chronic inflammation, which eventually leads to systemic tumor formation. In addition, TIAM2S-TG mouse represents a unique model to study the mechanistic progression from IBD to the development of colorectal carcinoma (CRC).

## 2. Results

### 2.1. TIAM2S-Overexpressing Mice Develop Spontaneous Tumors in Various Organs 

**Figure 1:**
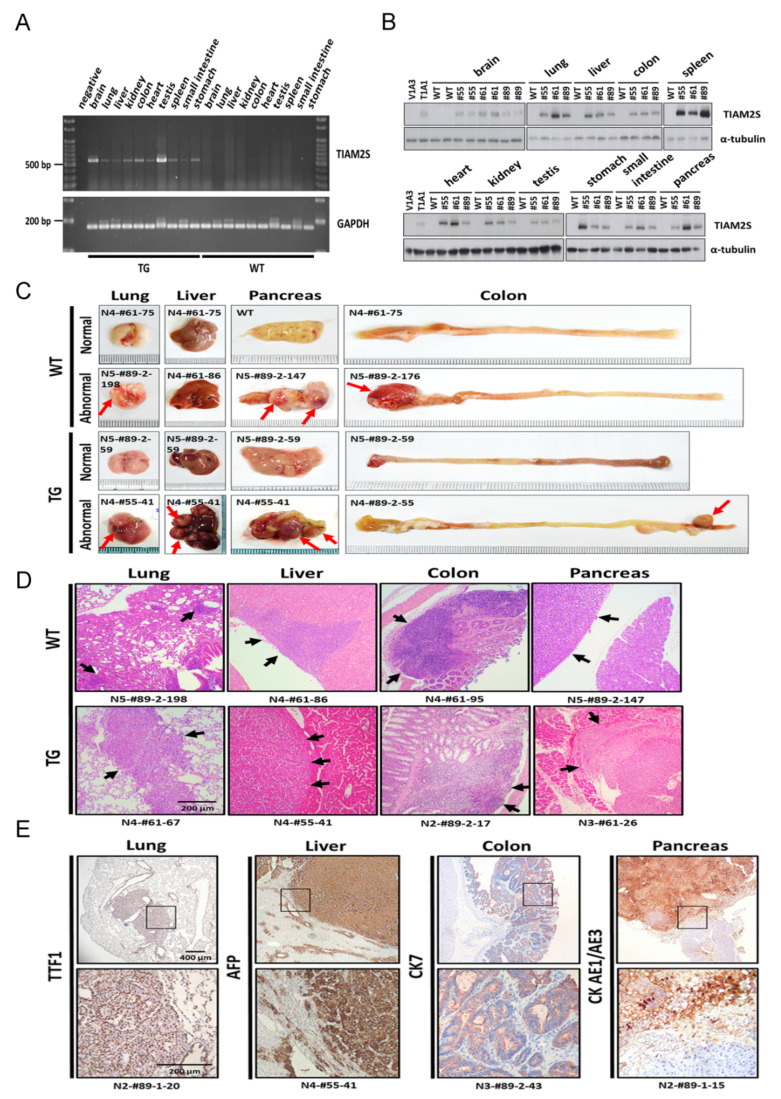
Human TIAM2S expression increases lymphocytic infiltration and tumor formation in mice. (**A**,**B**) Expressions of human TIAM2S mRNA (**A**) and protein (**B**) in various organs in the wildtype (WT) and transgenic animals were examined by RT-PCR and Western blot, respectively. Each organ from three derivative animal lines (#55, #61, and #89) was extracted. Recombinant human TIAM2S was recognized by anti-myc tag antibodies. GAPDH and α-tubulin were used as internal controls for mRNA (**A**) and protein (**B**). HepG2 stable clones, V1A3 (empty vector) and T1A1 (TIAM2S-expressing construct), were used as negative and positive controls for TIAM2S expression. (**C**) Representative images in mice at 2 years of age. Gross anatomy of normal and abnormal lung, liver, colon, and pancreas from WT and transgenic (TG) mice are shown. Red arrows indicate the areas of hypertrophy. Scale bars, 200 μm. (**D**) Representative histologic images of tissue sections of WT and TG mice by H&E staining show lymphocytic infiltration (black arrows) in the indicated organs and tissues. (**E**) Representative histologic images of immunochemical (IHC) staining indicating cancer markers in tissue sections of TIAM2S-TG mice (number indicates mice ID). Lower panel under each tissue sample indicates amplified images from the box marked region.

TIAM2S has been shown to act as oncogene in liver and lung cancer development. To characterize the systemic effect of TIAM2S on tumorigenesis, we generated pan-expression of TIAM2S mouse lines [9]. Five independent clones were identified by Southern blot analysis, and two female and one male F1 progeny were used to generate subsequent offspring. These (C57BL/6J) F2 to F5 mice were used in the experiments described here. The expression of human TIAM2S mRNA and protein in the wildtype (WT) and TIAM2S transgenic (TIAM2S-TG) mice were examined using reverse-transcriptase PCR (Figure 1A) and Western immunoblots (Figure 1B and Figure S1A), respectively. While expression of human TIAM2S is undetected in the wildtype mice (Figure 1A,B), various levels of TIAM2S protein were found in major organs from all three TG lines (Figure 1B). The data indicate that human TIAM2S is successfully expressed in TIAM2S-TG mouse lines. 

TIAM2S-TG mice were grossly normal at birth [9] and gain weight at about the same rate as the WT littermates up to 12 months of age. At the age of ~2 years old, several TIAM2S-TG mice showed abdominal masses. Twenty-four WT and fifty-three TIAM2S-TG mice with age ranked from 42 to 130 weeks were sacrificed and their organs were compared for the presence of abnormal cellular morphology. We found organomegaly occurred in major organs like lung, liver, colon, and pancreas in both WT and TG (Figure 1C). Histopathological analysis using Hematoxylin (violet) staining revealed lymphocytic infiltration in the major organs, demonstrating organomegaly in both WT and TG (Figure 1D) mice and were also noted other organs including the bladder, uterus, small intestine, and ovary only in the TG mice (Figure S1B). In addition, locations of lymphoid hyperplasia with close resemblance to tumors were observed in these enlarged organs. Specific antibody IHC stain confirmed tumor malignancies in the lung, liver, colon, and pancreas (Figure 1E).

The numbers of animals with abnormal lymphoid hyperplasia and development of tumors are summarized in Table 1. Overall, the tumor incidence of 45.3% (24/53) in the aged TG group was significantly higher than that of 12.5% (3/24) in the WT group (*p* < 0.005). Furthermore, while tumors in WT animals were found only in organs that are known to be associated with aged inflammation such as colon and pancreas, tumors in TG animals were found throughout the body, including lung, liver, colon, and pancreas, as well as kidney, ovary, and heart. In agreement with previous studies demonstrating TIAM2S oncogenic function in liver [6] and lung [7] cancer tumorigenesis, we observed the highest frequency of spontaneous tumor development in TIAM2S-overexpressing mice were lung (10 animals, 18.9%), followed by liver (6 animals, 11.3%) and colon (5 animals, 9.4%). Taken together, ectopic expression of human TIAM2S in the mouse demonstrated induction of lymphoid hyperplasia and promotion of tumor development.

### 2.2. Overexpression of TIAM2S Enhances AOM-Induced Colon Cancer Susceptibility 

**Figure 2:**
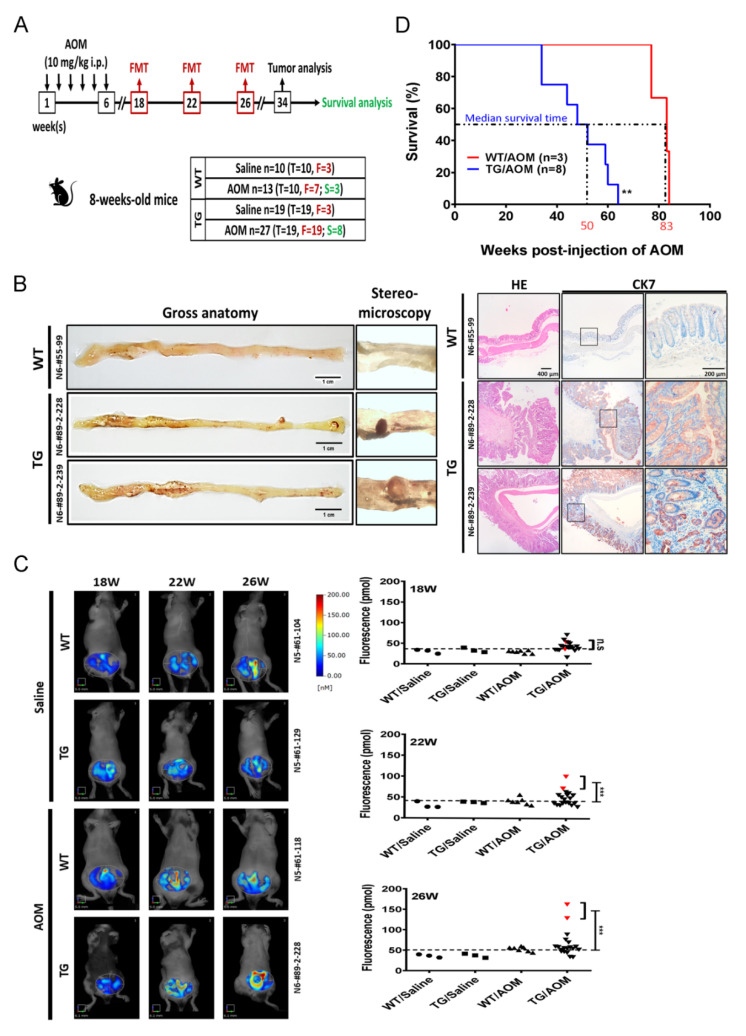
Ectopic TIAM2S expression increases the susceptibility to AOM-induced carcinogenesis. (**A**) Schematic representation of the experimental protocol of the AOM-induced colon cancer model. See Materials and Methods for a detailed description. The boxed number indicates weeks after starting the experiment/treatment. The black and red arrows specify time points for AOM injection and Fluorescence Molecular Tomography (FMT) monitoring, respectively. N: total number of animals used in each group, T: number of animals used for tumor assessment, F: number of animals used for FMT monitor, S: number of animals used for survival analysis. (**B**) Representative colonic images of WT and TG mice under AOM treatment. Examinations of gross anatomy and under stereomicroscopy (left), as well as H&E stain and IHC staining with CK7 are shown. Scale bar: 1 cm. (**C**). In vivo fluorescent tomographic imaging of mice under different treatments. Representative colonic field of chromatogram showed each experimental condition at the 18th, 22nd, and 26th weeks (left). Quantifications of fluorescent tomographic signals from animals under different treatments were plotted (right). Dot plots of three time points displaying the distribution of each animal’s signal. Red inverted triangle indicates two animals with tumor. The averaged signals from animals with tumor were compared to that from the normal animals at three time points. (**D**) The Kaplan–Meier survival curve was plotted and estimated median survival times of TIAM2S-TG (50 weeks) and WT (83 weeks) animals were compared. For all statistics, NS: not significant, ** *p* < 0.01, *** *p* < 0.001.

To further study the TIAMS-mediated tumorigenesis, we applied a well-established colon cancer model as a study platform [19] to test whether animals with TIAM2S-overexpression are more vulnerable to exogenous stimulation in the initiation of tumor development. Thirteen WT and twenty-seven TIAM2S-TG mice received weekly injections of azoxymethane (AOM, 10 mg/kg body weight), a carcinogenic agent that induces DNA damage via alkylation, for 6 weeks (Figure 2A). Ten WT and 19 TIAM2S-TG mice were treated in parallel with saline as the control group for this experiment. In vivo tracking of tumor formation was monitored by Fluorescence Molecular Tomography (FMT) Imaging in animals (Saline-WTx3, Saline-TGx3, AOM-WTx7, AOM-TGx19) via injection with IntegriSense^®^750 (PerkinElmer, Hopkinton, MA, USA )to conjugate integrin alpha v beta 3 (αvβ3) [20] and reconstitute body signals at 18, 22, and 26 weeks. Mice were sacrificed at week 34, and tumors were dissected from the animals for pathological evaluation except 3 and 8 mice from AOM-treated WT and TG groups, respectively, which were saved for survival evaluation. 

While none of the WT animals that underwent AOM treatment demonstrated tumor growth (0/10), two TIAM2S-overexpressing animals developed colonic adenoma (2/19) under the same treatment. To confirm the AOM-induced colonic adenoma, the phenotypes of two cases were represented by gross anatomy and stereomicroscopy (Figure 2B, left) and their cellular morphology was validated by IHC staining using anti-CK7 antibody (Figure 2B, right). 

Using a commercially available probe specifically designed to recognize integrin αvβ3, we reconstituted the biodistribution of fluorescent signals from FMT- monitored animals (Figure 2C, left panel) and found no difference between the quantified picomolecules from the two tumorous TG mice compared to the remaining group without tumors at 18 weeks (Figure 2C, right panel, top, *p* = 0.347). Nevertheless, significantly higher levels of integrin αvβ3 were detected in TG mice with tumor than those animals without tumors (Figure 2C, red triangles) at 22 (Figure 2C, right panel, middle, *p* < 0.001) and 26 weeks (Figure 2C, right panel, bottom, *p* < 0.001). The data demonstrated that integrin αvβ3 strongly increased and accumulated in the colonic regions of TIAM2S-TG mice compared to WT mice as early as 22 weeks after AOM treatment. Finally, we fed WT (N = 3) and TG (N = 8) mice up to 100 weeks to compare survival. The median survival time of the TG mice was 50 weeks and is much shorter than 83-week survival of the WT mice (*p* < 0.01, Figure 2D). Taken together, our data indicate that ectopic expression of human TIAM2S in mice increases the susceptibility of AOM-induced colonic adenoma and decreases the overall survival of animals. 

### 2.3. TIAM2S Expression Triggers Serotonin-Induced Immune Responses in Transgenic Mice

**Figure 3:**
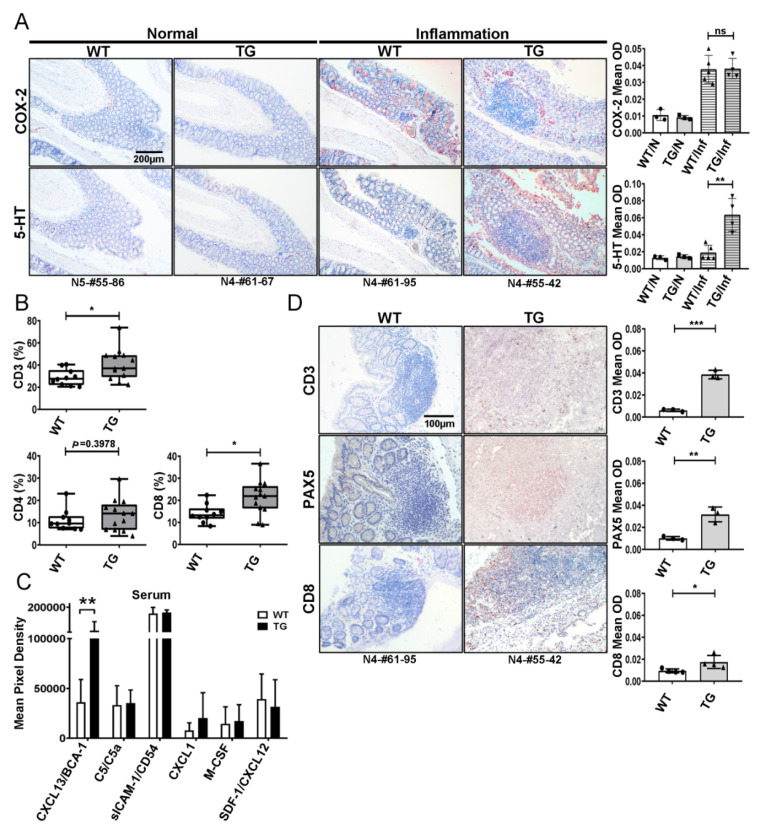
TIAM2S expression triggers serotonin-induced cellular immunity. (**A**) Representative histologic images of IHC staining of anti-COX-2 (upper panel) and anti-serotonin (lower panel) antibodies in colon sections of WT and TIAM2S-TG mice (number indicates mice ID). The levels of COX-2 and serotonin in different groups were approximated by the mean optical density (OD) quantified using Image J. (**B**) Summary data from fluorescence-activated cell sorting analysis show T lymphocyte distributions in the serum from WT and TG animals. Total and sub-population of T cells were recognized with anti-CD3 (upper panel), anti-CD4 (lower left), and anti-CD8 (lower right) antibodies. (**C**) Pro-inflammatory and inflammatory factors were profiled with serums from WT and TIAM2S-TG animals using cytokine and chemokine array and quantified signals of mean pixel density were compared. The level of CXCL13/BCA-1 was elevated in TG groups (*p* = 0.0097 by Student’s *t*-test). (**D**) Representative histologic images of inflamed colon sections from WT and TG mice were IHC stained with Anti-CD3 and -CD8 antibodies (T lymphocyte marker) and Anti-PAX5 antibody (B cell marker) to confirm the immune cell activation. For all statistics, NS: not significant, * *p* < 0.05, ** *p* < 0.01, *** *p* < 0.001.

Previous publications demonstrate that serotonin activates immune cells to produce pro-inflammatory mediators and results in gut inflammation (review see [21]). Our recent work revealed that TIAM2S functions as a novel regulator of cellular serotonin and that TIAM2S-TG mice exhibit higher serum serotonin levels than WT animals [9]. As TIAM2S-TG mice developed significantly higher proportions of infiltrative lymphocyte and tumors in various organs, we evaluated whether TIAM2S-mediated serotonin expression in the immune response may lead to inflammation profiles differing from those of aged WT animals. To this end, we first examined the expression of cyclooxygenase-2 (COX-2), an inducible form of the enzyme that catalyzes the first step in the synthesis of prostaglandins, associated with inflammatory diseases and carcinogenesis [22], and serotonin in the inflamed colons from both aged WT and TG mice (Figure 3A, left panels). In agreement with previous publications, we found minimally detected positive stains from the animals without inflammation, while inducible COX-2 was positively stained in inflamed regions of both WT and TG mice (*p* < 0.001, Figure 3A, right and upper panel), and there was no difference in the levels between these two groups (*p* = 0.967, Figure 3A, right and upper panel). Nevertheless, the quantified image data indicated that serotonin is expressed abundantly in the inflamed colon tissue of the TG mice, whereas that of WT mice maintained basal levels (*p* < 0.001, Figure 3A, right and bottom panel). These data suggest that human TIAM2S alters serotonergic homeostasis to modulate the colon microenvironment in the TG mice. 

Inflammation of the GI tract is characterized by the recruitment of activated innate and adaptive immune cells [21]. To profile TIAM2S-serotonin-prompted immune responses, we applied flow cytometry to analyze serum isolated from aged animals, which demonstrated that the proportion of total T lymphocytes as indicated by CD3+ cells is higher in TG mice compared to the WT animals (Figure 3B, top panel, *p* < 0.05). Further profiles of T lymphocyte subpopulations showed that TG mice support more CD8+ T cells (cytotoxic T cell, Figure 3B, bottom right, *p* < 0.05), but not CD4+ T cells (helper T cells, Figure 3B, bottom left, *p* = 0.398), compared to WT mice. In parallel, we assessed the profiles of cytokines and chemokines in peripheral serum from WT and TG mice to examine the possible TIAM2S-mediated stimulation pathway(s) in the circulatory system. While six cytokines/chemokines were detected in the serum of both groups, only CXCL13/BCA1 showed great enrichment in the TG animals (Figure 3C, *p* < 0.01). Collectively, these results suggest that TIAM2S overexpression elevates peripheral serotonin level and activates both T and B cell immune responses. 

To confirm the activated immune cells in the inflammatory GI sites, IHC staining was performed on organs. Although the differences were small and not significant (*p* = 0.21), a higher number of activated macrophages in the TG group were found (Figure S2A). We then performed IHC with anti-CD3, -CD8, -PAX5, and -CD45R antibodies to confirm the involvement of T lymphocytes and B cells in the composition of inflammatory tissues in the WT and TG mice (Figure 3D and Figure S2B). The quantified results confirmed significantly higher numbers of CD3+ (Figure 3D, top panel, *p* < 0.001) and CD8+ (Figure 3D, bottom panel, *p* < 0.05) T cells enriched in TG colons. In addition, enhanced signal for PAX5+ (Figure 3D, middle panel, *p* < 0.01) and CD45R+ cells (Figure S2B, *p* < 0.001) were obtained. Taken together, the data demonstrate consistent evidence from peripheral blood to solid organs that human TIAM2S expression promotes elevated serotonin levels, expands the CD8^+^ T cell subset in PBMCs, and elevates the serum level of CXCL13/BCA-1. In turn, aggregated T lymphocytes and B cells are recruited to the site of inflammation of the GI tract which results in diffuse lymphoid infiltration and tumor development.

### 2.4. TIAM2S Initializes Colonic Chronic Inflammation 

**Figure 4:**
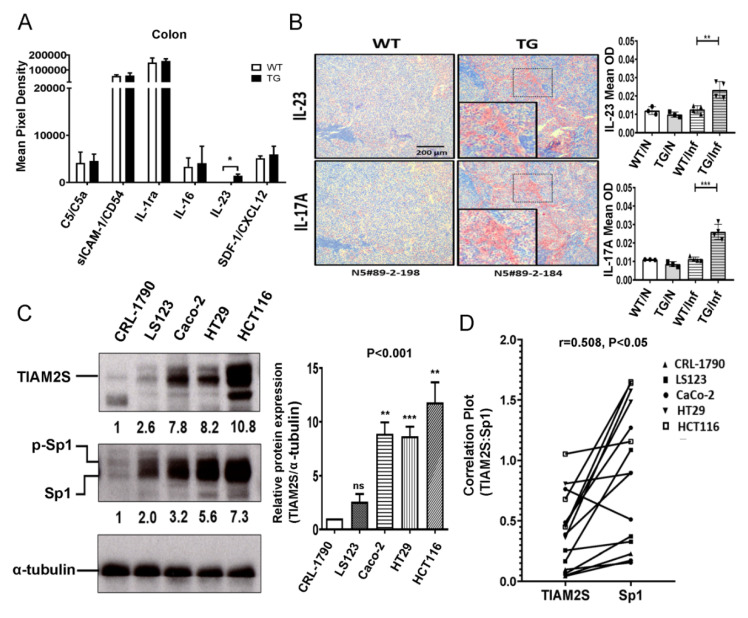
TIAM2S enhances chronic colonic inflammation as a human IBD model. (**A**) Inflammatory cytokine and chemokine profiles of the colon from WT and TIAM2S-TG mice were examined and compared using cytokine and chemokine array. The level of IL-23 was increased in TG groups (*p* = 0.0305 by Student’s *t*-test). (**B**) Representative images of colonic Tertiary Lymphoid Organs (TLOs) were IHC stained with anti-IL23 (upper panel) and -IL-17A (lower panel) antibodies from WT and TG mice with different inflammatory states (number indicates mice ID). Expression levels of IL-23 and IL-17A were increased in the inflammatory TG mice (IL-23, *p* < 0.01; IL-17A, *p* < 0.001 by one-way ANOVA with Bonferroni’s multiple comparison test). (**C**). Representative Western blot image shows the expression of TIAM2S and SP1 in various colon cancer cell lines (Left). The level of α-tubulin was used as loading control. Quantification of TIAM2S and SP1 signal intensities show significantly increased in colorectal carcinoma (CRC) cell lines in comparison with the normal CRL1790 cells (right). (**D**). Pearson correlation analysis shows significant association of TIAM2S and SP1 expressions in various CRC cell lines. For all statistics, NS: not significant, * *p* < 0.05, ** *p* < 0.01, *** *p* < 0.001.

To characterize the downstream signaling in response to TIAM2S-mediated immune cell activation, the profiles of cytokine/chemokine interactions in colonic tissues were studied. As demonstrated in Figure 4A, expression of IL23 is undetectable in WT animals but was significantly elevated in TG mice (Figure 4A, *p* < 0.05). 

The heterodimer IL-23 formed by p19 and p40 plays a major role in inflammatory diseases, such as multiple sclerosis (MS) and IBD [23]. We hypothesized that IL-23 activates CD8+ T cells to express IL-17A and serves as a pro-inflammatory factor with ectopic TIAM2S expression. To test this hypothesis, we performed IHC staining in the Tertiary Lymphoid Organs (TLOs), the local nodule formed by the interaction between lymphocytes and the tissue-resident stromal cells [24] under chronic inflammation. It was found that the accumulated IL-23/IL-17A axis located in colonic TLOs from TG mice (Figure 4B, left), and the presence of positive cell staining are both elevated in inflamed colonic tissues from the TG mice compared to the WT mice (Figure 4B, right, IL-17A, *p* < 0.001; IL-23, *p* < 0.001). Collectively, ectopic TIAM2S expression promotes chronic inflammation through an increase in serotonin level and triggers CD8+ T lymphocytes to develop IBD through the interleukin (IL)-23/IL-17 axis [9]. 

To link the murine TIAM2S-mediated tumorigenesis to humans, it is important to show that neuron-specific TIAM2S expression is present in human colorectal carcinoma cells as well. Our data demonstrated increased enrichment of TIAM2S proteins in malignant CRC cell lines (ranging from 2.24- to 9.35-fold) compared to CRL-1790 (Figure 4C, left and top panel, *p* < 0.01), which are normal human fetal colon epithelial cells. In addition, the expression of SP1 (Figure 4C, left and middle panel, *p* < 0.001), the transcription factor controlling ectopic TIAM2S expression in hepatocellular carcinoma [10], is highly correlated with the TIAM2S protein in these CRC cell lines (Figure 4D, r = 0.508, *p* < 0.05). Collectively, these results confirm SP1 controls ectopic expression of TIAM2S in human malignant, non-neuron cells. Finally, we propose a working model for TIAM2S mediated development of CRC.

## 3. Discussion

Although our previous study demonstrated that TIAM2S is able to trigger tumor growth and invasion in vivo [6], the immune-deficient nude mice model was unable to elucidate the role of TIAM2S in the modulation of immunity in animals. To overcome this limitation, we have successfully generated transgenic mice specifically overexpressing human TIAM2S. The TIAM2S-TG mice were fertile and without observable detrimental deficits, thus providing an excellent animal model to investigate TIAM2S-mediated physiological and pathological effects in vivo [9]. In the current study, we found that aged TG animals, in comparison with the WT mice of the same age, exhibit significantly elevated tumor incidence and lymphocytic infiltrations in various organs, including colon, liver, and lung. Furthermore, while WT mice were unable to develop colon cancer under AOM treatment due to colon cancer susceptibility locus 3 (Ccs3) resistance in the C57B6/JNarl strain background [25], the carcinogen alone effectively induced development of colonic adenoma in TG mice starting at 22 weeks after AOM treatment. These observations demonstrated that TIAM2S-TG mice are primed to develop tumors under carcinogen induction and aging. Further characterization of the inflamed tissues from both WT and TG mice found that TIAM2S-mediated systemic inflammation is associated with elevated serotonin levels, aberrant immune cell populations, and pro-inflammatory cytokine profiles. Thus, TIAM2S-serotonin-mediated chronic inflammation is distinct from age-associated immune responses and contributes to tumorigenesis.

Our recent study demonstrated for the first time that TIAM2S is a novel regulator for serotonin level and therefore may shape brain neuroplasticity and affect locomotion behavior [9]. Serotonin is both a neurotransmitter and a hormone involved in the regulation of various physiological functions by its actions in the central nervous system (CNS) and in peripheral organ systems. Peripheral serotonin, predominantly produced by enterochromaffin cells of the GI tract, is known to affect various immune cells [16] and lead to inflammation [17]. Furthermore, the vast majority of total peripheral serotonin is stored in platelets and released upon platelet activation in environments like sites of thrombosis or an inflammatory field [26]. Consequently, serotonin modulates several immunological events, such as chemotaxis, leukocyte activation, proliferation, cytokine secretion, energy, and apoptosis, plays pivotal roles in the clinical outcome of diseases such as major depression [27] and inflammatory bowel disease [28], and contributes to the development of various cancers, including breast [29], liver [30], pancreatic [31], and colon [11] cancers. Although studies have reported that serotonin activates downstream targets of PI3K-AKt-mTOR and Jak1-STAT3-ERK1/2 to promote glycolysis and growth in breast [29], liver [30], and pancreatic ductal adenocarcinoma [31] tumors, the role of serotonin-induced immune response in the lesion site (tumor micro-environment) is largely unexplored. In the current study, we found elevated serum and colonic serotonin in aged TIAM2S-TG mice, which was associated with TIAM2S-, but not age-mediated inflammation. High serum serotonin in the TIAM2S-TG mice activates both T and B lymphocytes and lymphocyte chemoattractant CXCL13/BCA-1 in the peripheral circulation. In turn, the CD3+/CD8+ T cells are attracted to the colonic lesion sites and trigger IL23 and IL17A expression. Previous studies reported that the naïve B cells and T lymphocytes utilized CXCL13/BCA-1, which is mainly produced by follicular dendritic cells, monocytes, and macrophages [32], for migration, homing, and maturation [33,34], as well as for the formation of TLOs [31] in chronically inflamed tissues. Our data support this notion and demonstrated that ectopic TIAM2S expression exacerbates the immune response and leads to conditions resembling chronic inflammatory disease.

Inflammatory Bowel Disease is a chronic relapsing inflammation of the GI tract resulting from a dysregulated mucosal immune response to environmental factors in genetically susceptible hosts [35]. The pathophysiology of IBD is multifactorial and not completely understood, but genetic, environmental, microbial, dysregulated immune responses, oxidative stress, and inflammatory mediators are known to be involved [36]. Murine IBD models have been successfully generated, and these animal models were used to understand the pathogenic mechanisms of human disease and evaluate potential treatments of IBD [37]. However, no animal model has fully reproduced the clinical and histopathological features of human IBD. Previous studies indicate that idiopathic IBD is caused by cytokine-driven, non-infectious inflammation of the gut. For example, Crohn’s disease (CD) is associated with excessive IFN-γ/IL-17 and IL-12/IL-23 production, while ulcerative colitis (UC) is associated with excess IL-13 [38]. We found an increase of IL-23 in TIAM2S-TG mice at one year of age that supported a chronic inflammation profile. Although the IL-23/IL-17 axis is known as an essential pathway of CD4+Th17 cells in the IBD model [39] and validated with experimental models of colitis [21,40], our data demonstrated that TIAM2S-mediated IL-23 activation results in IL-17 secreting CD8+ T cell expansion, which also plays a pathogenic role in chronic colonic inflammation. In fact, CD8+ T cells possess pathogenic plasticity via IL-23/IL-17 signaling also has been reported in chronic obstructive pulmonary disease [41] and IBD [19,42,43]. Furthermore, an IBD model with an induced TNF defect in posttranscriptional regulation showed CD8+ T cells functions as IBD effector [44,45]. Altogether, our TIAM2S transgenic mouse is a novel model for elucidating the pathogenesis of IBD in relation to native CD8+ T cell population and expression during chronic inflammation.

Currently available IBD treatments can decrease the duration of active disease; however, the medications are with significant adverse effects. Thus, the search for novel therapeutic strategies that can restore intestinal homeostasis remains ongoing. On the other hand, the crucial roles of serotonin in numerous immunological processes have offered promising treatment modalities through the use of psychedelics. These classical psychoactive substances, which exert psychopharmacological activity, have been attempted as therapeutics for various diseases with a chronic inflammatory etiology, including autoimmune and chronic inflammatory conditions, infections, and even cancers [46]. For example, tryptophan treatment attenuates the symptoms and severity of DSS-induced colitis [47] and reduces gut permeability, expression of pro-inflammatory cytokines, and increases expression of the apoptosis initiators caspase-8 and Bax [48]. Thus, tryptophan may be considered as a promising candidate for the treatment of IBD.

Great progress has been made in the past decade to increase our understanding of the link between chronic inflammation and cancer development. It is now evident that the common factor among carcinogenic agents is the activation of genes controlling inflammatory cell-signaling pathways and that, in turn, these signals control all aspects of the cancer process [49]. Based on our prior and current studies, we discovered that TIAM2S provokes a pro-inflammatory immune microenvironment permissive to colorectal tumorigenesis. TIAM2S induced the expression of pro-inflammatory cytokines that are associated with the recruitment of tumor-infiltrating lymphocytes in the tumors. These effects are likely mediated by serotonin that directly activates immune cells and triggers various immune responses. Therefore, pro-inflammatory stimulation and selective enrichment of cytotoxic T-cells represents an alternative mechanism by which TIAM2S facilitates CRC tumorigenesis. Nevertheless, TIAM2S expression in our murine model alters cellular serotonin homeostasis and results in chronic inflammation, providing a unique animal model to study chronic GI inflammation-related tumorigenesis.

## 4. Materials and Methods

### 4.1. Animals and Colon Cancer Cell Lines

The wildtype (WT) and human TIAM2S-transgenic (TG) mice with the C57BL/6JNarl genetic background were used in this study. The establishment of TG mice was described in our article [9]. Three distinct lines of TG mice (Lines #55, #61, and #89) were maintained in the pathogen-free laboratory animal center in National Cheng Kung University (NCKU) under a 12-h light/dark cycle and a constant temperature and humidity. The study was approved by the Animal Ethics Committee at National Cheng Kung University, and all experiments applied to the animals complied with the relevant regulatory standards.

To examine the expression of TIAM2S in colorectal carcinoma (CRC) cells, CRL-1790, LS123, Caco-2, HT-29, and HCT116 5 CRC cell lines were used. Cells were routinely maintained according to the guidelines from ATCC (https://www.atcc.org/).

### 4.2. Spontaneous and Azoxymethane-Treated Mouse Models

Twenty-four WT and fifty-three TG mice were grown to 2 years old in pathogen-free cages. Phenotypically, enlarged abdomens were observed in some of the aged animals. To characterize the abnormalities in the mice, organs were dissected from animals, including brain, bladder, colon, small intestine, liver, spleen, heart, lung, kidney, pancreas, stomach, ovary, uterus, and testis. These organ samples were divided into two parts, one for protein extraction and the other for tissue section. The pathological conditions of studied animals were classified and histologically confirmed by pathologists.

To study the susceptibility to azoxymethane (AOM, A5486; Sigma Aldrich, St Louis, MO, USA)-induced colon tumorigenesis under TIAM2S expression, a total of 69 8-week-old animals (23 WT and 46 TIAM2S-TG) were used in the AOM-induced colon carcinogenesis mouse model. AOM working solution (10 mg/kg body weight) was injected into mice (13 WT and 27 TIAM2S-TG) once a week for 6 weeks, while the control mice (10 WT and 19 TIAM2S-TG) received a sterile isotonic saline injection. At the 18th, 22nd, and 26th weeks after AOM injection, randomly assigned animals (3 WT-Saline, 7 WT-AOM, 3 TG-Saline, and 19 TG-AOM) were monitored for tumor development using FMT with IntegriSense^®^750. Animals were sacrificed for tumor assessment at 34 weeks after injection. Three and eight AOM-treated WT and TG animals, respectively, were followed for survival testing.

### 4.3. In Vivo Fluorescence Molecular Tomography (FMT)

AOM-treated animals were randomly selected to track tumor formation using in vivo fluorescence tomographic imaging (FMT) at the 18th, 22nd, and 26th weeks after AOM injection. Before FMT imaging, animals were depilated and injected with IntegriSense™ 750 (NEV10873, PerkinElmer, Hopkinton, MA, USA) 2 nmol in 150 μL per animal via tail vein to circulate throughout the whole body for 24 h. The mice were anesthetized with 2% isoflurane and placed in a cassette for image using FMT 4000™ fluorescence tomography in vivo imaging system (PerkinElmer, Hopkinton, MA, USA). Images were acquired by FMT 4000 at 750 nm wavelength. The resulting 3D dataset was reconstructed, and the region of interest (ROI) was defined encompassing the colon tissue of the abdominal area using the software TrueQuant Imaging Software (7005319, PerkinElmer, Hopkinton, MA, USA) with a set detection threshold at 30% of the max concentration within the ROI. The quantified amount of fluorochrome in the ROI was calculated by TrueQuant to present in picomole.

### 4.4. RT-PCR Analysis

Approximately 2 μg of total RNA isolated from mice tissue using TRIzol reagent was reverse transcribed into cDNA by High Capacity cDNA Reverse Transcription Kit (4368814, Applied Biosystems, Foster City, CA, USA). The expression of TIAM2S transcripts was examined by PCR to amplify 352-bp amplicons using GoTaq DNA polymerase (M3008, Promega, Madison, WI, USA) with its buffer and dNTP (forward primer 5′-CTTCTGCCCCATTAAACGAA-3′, reverse primer 5′-CAACAGATGGCTGGCAACTA-3′).

### 4.5. Protein Extraction and Immunoblotting

Tissues/organs were excised to about 100 mg to embed in 1 mL of RIPA buffer (50 mM Tris-HCl, pH 7.4, 150 mM NaCl, 1 mM EDTA, 1% NP-40, 0.5% sodium deoxycholate, 0.1% SDS, 1% Triton X-100, 100 mM PMSF, 1M DTT and a cocktail of protease inhibitors), homogenized, and centrifuged at 4 °C with 19,600× *g* for 20 min, and the supernatant was harvested. Approximately 100 μg of total lysate was loaded onto 8% SDS-PAGE. The lysates of colon cancer cells were directly isolated by RIPA buffer, and ~40 μg of cell lysates was loaded to run 8% SDS-PAGE. The separated proteins were transferred onto a polyvinylidene difluoride (PVDF) membrane treated with methanol and blocked with 5% bovine serum albumin in TBST (10 mM Tris-HCl, pH8.0, 150 mM NaCl, and 0.05% Tween 20) for at least 1 h. The membranes were probed with anti-TIAM2 (1:200, SC-13304, Santa Cruz, CA, USA) or anti-TIAM2 (1:1000, abcam, Cambridge, UK), and anti-Myc tag antibodies (1:1000, 2272S, Cell Signaling Technology, Danvers, MA, USA) overnight and then donkey anti-goat IgG-HRP (1:5000, SC-2020, Santa Cruz, CA, USA) and Goat Anti-Rabbit IgG H&L (1:20000, ab6721, Abcam, Cambridge, UK) for 2 h in the next day, respectively. Re-probing membrane with anti-α-tubulin (1:4000, 2125S, Cell Signaling) was used as the loading control. Signal detections were performed using the enhanced chemiluminescence (ECL) detection system (NEL105001EA, Western Lightning Plus-ECL, PerkinElmer, Hopkinton, MA, USA).

### 4.6. Flow Cytometry

Peripheral blood of mice was harvested by cardiac puncture technique after euthanasia into a tube containing heparin as anti-coagulant. Samples were diluted with saline in an equal volume and centrifuged at 400× *g* for 10 min at room temperature. After removal of upper plasma, the Ficoll^®^ Paque Plus buffer (17-1440-02, GE Healthcare Life Sciences, Shanghai, China) were mixed with the cell resuspension with saline in a 50-mL tube. After centrifugation at 800× *g* for 30 min at room temperature, the samples were separated into layers from top to bottom as platelet in plasma, peripheral blood mononuclear cells (PBMC), Ficoll buffer, and red blood cells. The upper layer of a sample was aspirated carefully and then collected PBMC composed of lymphocytes and monocytes predominantly. To count the frequencies of T lymphocytes by BD FACSCalibur™ platform (BD Biosciences, Taipei, Taiwan), cells were labeled with PE Hamster anti-CD3e (553063, BD Biosciences), APC anti-CD4 (17-0042, eBioscience, Thermo Fisher Scientific Inc., Waltham, MA), and FITC anti-CD8a (11-0081, eBioscience) to distinguish a subpopulation of T lymphocytes.

### 4.7. Hematoxylin-Eosin Staining, Immunohistochemistry, and Immunofluorescence

Tissues/organs were fixed with formalin and embedded in paraffin. The paraffin sections were treated with xylene for deparaffinization, followed by 95% alcohol for 1 min, 80% alcohol for 30 s, 70% alcohol for 30 s, and water as a final treatment for rehydration. The tissue sections were stained in hematoxylin solution for 12 min and washed in running tap water for 15 min, followed by staining in eosin solution for 2 min. Dehydrating through 95% alcohol for 10 s, absolute alcohol for 20 s twice, and the three times of xylene for 2 min, the sections were mounted with a xylene-based mounting medium.

For immunohistochemistry (IHC), the tissue sections proceed with deparaffinization and rehydration were applied with the avidin-biotin-peroxidase complex system (LSAB+ Dako REAL Detection Systems, K5003, DaKoCytomation, Glostrup, Denmark) in combination with anti-CK7 (ab181598, Abcam, Cambridge, UK), anti-TTF1(ab76013, Abcam, Cambridge, UK), anti-CD45R (14-0452-81, RA3-6B2, eBioscience, Thermo Fisher Scientific Inc., Waltham, MA), anti-CD3 (GTX16669, Genetex, Irvine, CA, USA), anti-PAX5 (GTX54532, Genetex, Irvine, CA, USA), anti-serotonin (S5545, Sigma-Aldrich), and anti-COX2 (160112, clone CX229, Cayman Chemical, Ann Arbor, MI, USA) antibodies and developed chromogen by AEC+ Substrate-Chromogen (K3461, Dako). For immunofluorescence (IF), the colon sections were incubated with proteinase K to increase antigen retrieval, blocked with 5% bovine serum albumin in PBS for an hour at RT, and incubated with anti-F4/80 (GTX26640, Genetex) at 4 °C overnight. After washing with PBS, slides were incubated with Goat Anti-Rat IgG H&L (Alexa Fluor^®^ 488, ab150157, Abcam). Nuclei were stained with 4, 6-diamidino-2-phenylindole, dihydrochloride (DAPI, D1306, Invitrogen, Carlsbad, CA, USA) in blocking solution for 5 min at RT and covered with mounting medium.

To quantify the signal intensity, 10 fields were randomly selected per section from each individual animal and measured using Image J (https://imagej.nih.gov/ij/) incorporated with a plugin “colour_deconvolution” [47]. The values were transformed into optical density by the formula equation OD = log_10_ (1/T), T representing the value from Image J.

### 4.8. Profile of Cytokine and Chemokine Secretion

To measure cytokine/chemokine production, serums and colon tissue were collected from matched mice in all mouse lines. The blood was isolated from animals by cardiac puncture after sacrifice. After standing for 20 min, the serum (supernatant) was transferred into a new tube and stored at −80 °C. Subsequently, colon was dissected from animal and lysate was extracted for further analysis. To analyze cytokine and chemokine secretion, 200 μL of serum or 300 μg of colon lysates was assayed using a Mouse Cytokine Array Panel (ARY006, R&D system, Inc., Minneapolis, MN, USA). The array images were exposed by X-ray films and quantified from at least three independent experiments by the Protein Array Analyzer programmed in Image J.

### 4.9. Statistical Analysis

Data were analyzed by Student’s *t*-test or one-way ANOVA followed by Tukey’s multiple test using GraphPad Prism 5.0 (GraphPad Software, La Jolla, CA, USA). Kaplan–Meier estimator was used for survival analysis. All data are presented as mean ± S.D., and the significance was set at *p* < 0.05. For all tests, significance was considered at *p*-value < 0.05.

## 5. Conclusions

We established a murine model to overexpress human TIAM2S and demonstrated that ectopic TIAM2S expression results in colonic inflammation through serotonin-mediated immune responses. We found that intestinal pro-inflammatory IL-23 activated IL-17 secretion in CD8^+^ T cells, leading to colonic inflammation and tumor formation. Elevated serotonin levels, activated lymphocyte and cytokine/chemokine profiles observed in the TIAM2S-TG mice resemble the condition of human inflammatory bowel disease and elucidate a possible mechanism leading to colonic tumor development.

## Figures and Tables

**Table 1:** Spontaneous tumor incidence in WT and TG mice ^a.^

	WT	TG
Age Wks (Median)	42–126 (99)	50–130 (105)
Total number (N)	24	53
Animal with tumor (N)	3	24 ^b^
Liver	0	5
Lung	0	9
Colon	2	3
Pancreas	1	3
Small intestine	0	3
Kidney	0	2
Spleen	0	1
Ovary	0	1
Stomach	0	0
Bladder	0	0
Rectum	0	0
Heart	0	1
Testis	0	0
Uterus	0	0

^a^ The overall tumor incidence was significantly different between WT and TG groups (*p* < 0.005); ^b^ There are 4 animals which developed tumors in multiple organs and tissues.

## Supplementary Materials

The following are available online at https://www.mdpi.com/2072-6694/12/7/1844/s1, Figure S1: TIAM2S-transgenic mice model used in this study. Figure S2: Expression of macrophages and CD45R in inflammatory nodules of TG and WT animals.
