# Supplementary material for: TIAM2S Mediates Serotonin Homeostasis and Provokes a Pro-Inflammatory Immune Microenvironment Permissive for Colorectal Tumorigenesis
Source: Cancers (Basel). 2020 Jul 8;12(7):1844. doi: 10.3390/cancers12071844 (PMC7408714; doi:10.3390/cancers12071844)
Supplement: Supplementary file 1 [file cancers-12-01844-s001.pdf]

Supplementary Materials: TIAM2S Mediates Serotonin Homeostasis and Provokes a Pro-Inflammatory Immune Microenvironment Permissive for Colorectal Tumorigenesis

Ya-Ling Chan [1], Wei-Chung Lai [2], Jia-Shing Chen [3], Joseph Ta-Chien Tseng [1], Pei-Chin Chuang [4], Jonathan Jou [5], Chung-Ta Lee [6] and H. Sunny Sun [2,*]

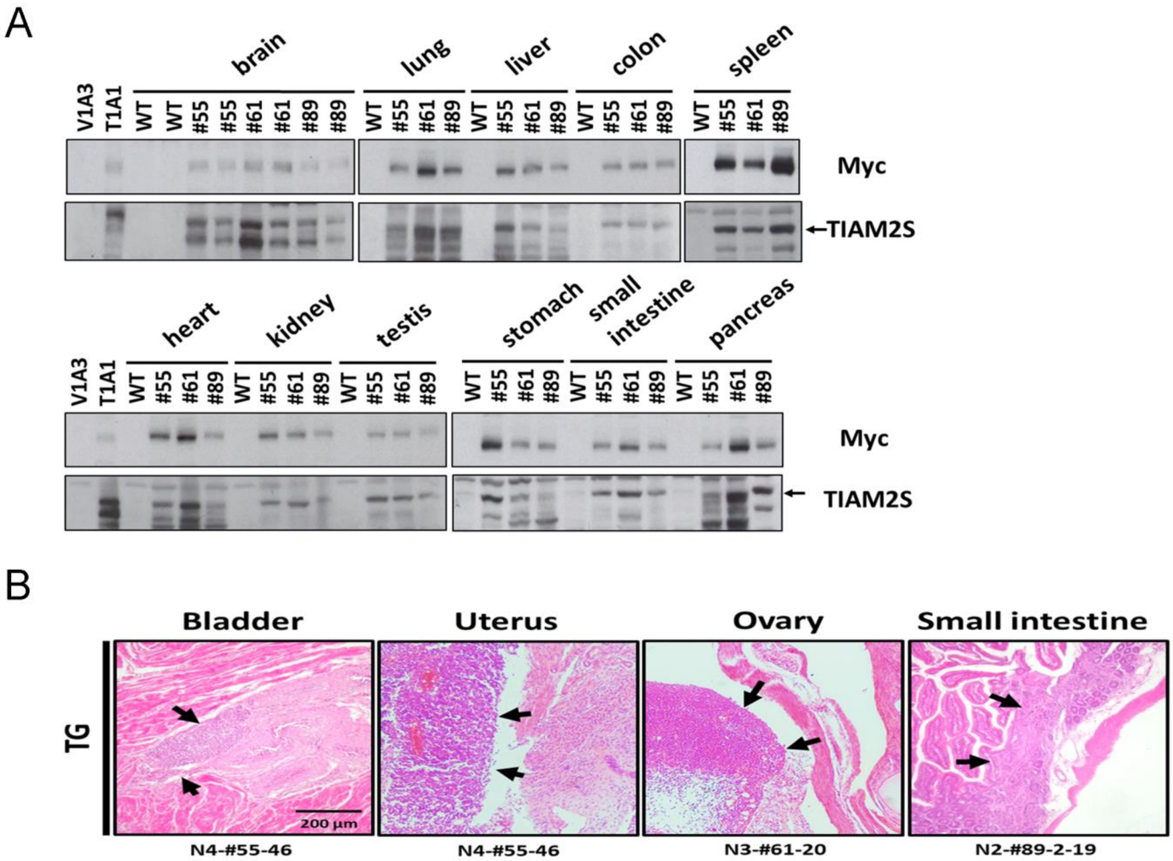

Figure S1. TIAM2S transgenic mice model used in this study. (A) Expression of human TIAM2S in various mouse tissues/organs were detected using anti-Myc (upper panel) and anti-TIAM2S (lower panel) antibodies. (B) Lymphocytic infiltration in bladder, uterus, ovary, and small intestine of TG mice. Black arrows indicate the lymphocytes (violet).

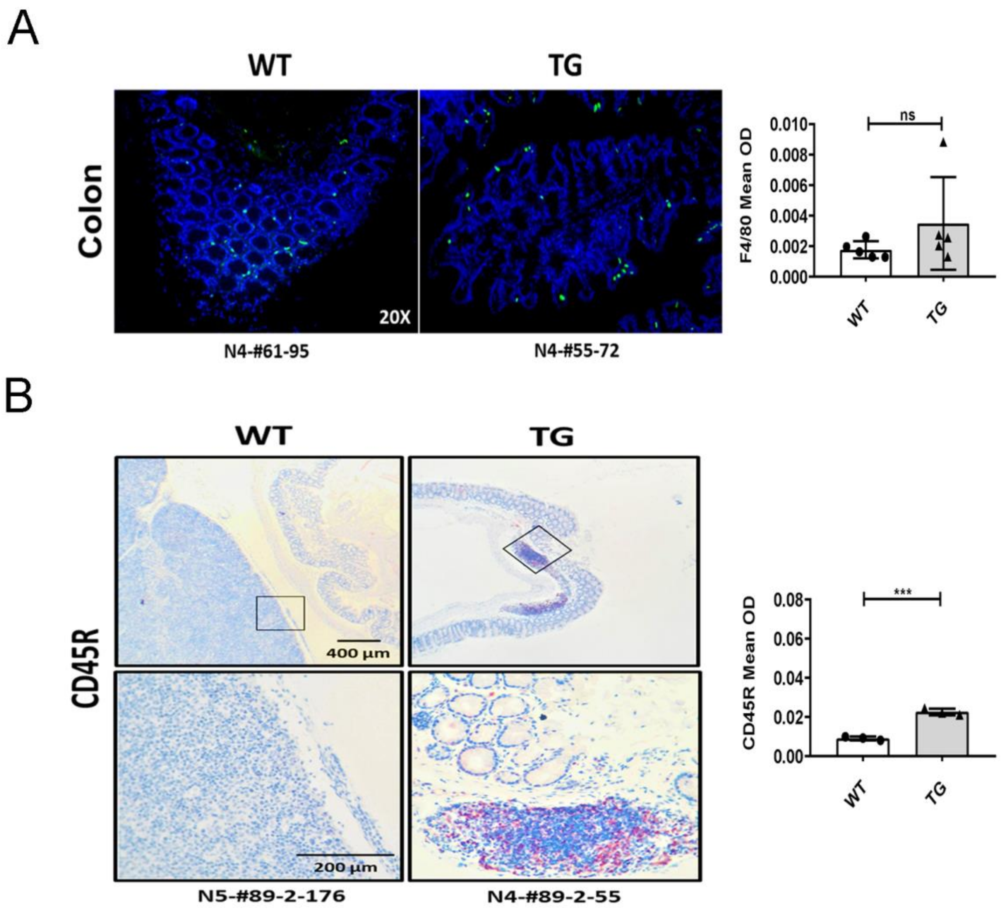

Figure S2. Expression of macrophages and CD45R in inflammatory nodules of TG and WT animals**.** (**A**) IF staining of inflamed colon sections with anti-F4/80 antibody (left) and reveal no differences between these two groups (right). (**B**) IHC staining of inflamed colon sections with anti-CD45R antibody (left) to confirm B cell activation (right). For all statistics, NS: not significant, *: $P < 0.05$, **: $P < 0.01$, ***: $P < 0.001$.
